# Activity of phytochemical constituents of *Curcuma longa* (turmeric) and *Andrographis paniculata* against coronavirus (COVID-19): an in silico approach

**DOI:** 10.1186/s43094-020-00126-x

**Published:** 2020-10-16

**Authors:** Kalirajan Rajagopal, Potlapati Varakumar, Aparma Baliwada, Gowramma Byran

**Affiliations:** Department of Pharmaceutical Chemistry, JSS College of Pharmacy (A constituent college of JSS Academy of Higher Education & Research-deemed University), Ooty, The Nilgiris, Tamilnadu 643001 India

**Keywords:** Coronavirus (COVID-19), *Curcuma longa* (turmeric), *Andrographis paniculata*, Docking studies, MM-GBSA

## Abstract

**Background:**

In early 2020, many scientists are rushing to discover novel drugs and vaccines against the coronavirus, and treatments for COVID-19, because coronavirus disease 2019 (COVID-19), a life-threatening viral disease, affected first in China and quickly spread throughout the world. In this article, in silico studies have been performed to explore the binding modes of chemical constituents for natural remedies like *Curcuma longa* (turmeric) and *Andrographis paniculata* against COVID-19 (PDB ID 5R82) targeting coronavirus using Schrodinger suit 2019-4. The molecular docking studies are performed by the Glide module, in silico ADMET screening was performed by the QikProp module, and binding energy of ligands was calculated using the Prime MM-GB/SA module.

**Results:**

The chemical constituents from turmeric like cyclocurcumin and curcumin and from *Andrographis paniculata* like andrographolide and dihydroxy dimethoxy flavone are significantly binding with the active site of SARS CoV-2 main protease with Glide score more than − 6 when compared to the currently used drugs hydroxychloroquine (− 5.47) and nelfinavir (− 5.93). When compared to remdesivir (− 6.38), cyclocurcumin from turmeric is significantly more active. The docking results of the compounds exhibited similar mode of interactions with SARS CoV-2. Main protease and the residues THR24, THR25, THR26, LEU27, SER46, MET49, HIE41, GLN189, ARG188, ASP187, MET165, HIE164, PHE181, and THR54 play a crucial role in binding with ligands.

**Conclusion:**

Based on in silico investigations, the chemical constituents from turmeric like cyclocurcumin and curcumin and from *Andrographis paniculata* like andrographolide and dihydroxy dimethoxy flavone, significantly binding with the active site of SARS CoV-2 main protease, may produce significant activity and be useful for further development.

## Background

Coronavirus disease 2019 (COVID-19) is a life-threatening disease which was affected first in China and quickly spread throughout the world [1–6]. According to the WHO data, as of the second week of April 2020, there are 21.5 lakhs peoples in the world affected by COVID-19, out of these more than 1.5 lakhs peoples died. With more asymptomatic infections being found among COVID-19 cases, it is worthy of consideration the detailed current evidence and understanding of the transmission of SARS CoV, MERS-CoV, and SARS CoV-2 and discussion on pathogen inactivation methods on coronaviruses is very important [7–12].

In this emergency situation, it is very difficult to discover novel drugs with all clinical trials and also determine the side effects, adverse effects, etc. So, it is important to find some natural remedies for the prevention and treatment of COVID-19. From the literatures, the natural products like *Curcuma longa* (turmeric) and *Andrographis paniculata* were reported for various biological activities and used traditionally for curing many diseases. Also, there is no or minimum side effects reported when compared to allopathic drugs.

The dried and powdered root *Curcuma longa* (turmeric) is belonging to the Zingiberaceae family, which is being cultivated in many countries worldwide. It has many uses such as textile dyes, herbal medicines, or food products. The biological properties of its chemical components were reported for inhibition of platelet aggregation [13], anti-diabetic [14], anti-tumor [15–17], anti-inflammatory effects [18], antioxidant effects [19], anti-platelet aggregation effects [20], gastro-protective effects [21], lipid-lowering effects [22], Alzheimer's effects [23], etc.

*Andrographis paniculata* was reported for the treatment of liver diseases [24], fever, common cold [25], acute diarrhea [26], hypertension [27], chicken pox, leprosy [28], hepatitis [29], malaria [30], anti-inflammatory effects [31], anti-cancer [32], diabetes [33], etc.

As part of our ongoing research on searching the potent biological molecules against various diseases by in silico and wet lab methods [34–44], we have designed and evaluated various heterocyclic compounds for their biological activities. Using different modules (Glide, QikProp, and Prime) of Schrödinger suite LLC various computational methods like molecular docking, ADMET screening, and binding-free energy, calculations were performed to find the interactions responsible for SARS CoV-2 main protease inhibition. These studies will provide the requirement of key structural features in the design of potential drug candidates.

## Methods

The 3D crystal structure of COVID-19 protein called SARS CoV-2 main protease receptor co-crystallized with 6-(ethylamino) pyridine-3-carbonitrile (PDB ID 5R82, resolution 1.31 Å) was retrieved from the protein data bank. The protein was prepared using the protein preparation wizard of epic module of Schrödinger suite 2019-4. The protein structure retrieved from the RCSB protein data bank is a monomer with co-crystallized ligand. The protein was prepared by using the protein preparation wizard by refining bond orders, addition of hydrogens, and deleting water molecules beyond 5 Å, and missing chains are included by using the Prime module [45] of Schrödinger suite 2019-4. Protein minimization was performed using optimized potentials for liquid simulations (OPLS3) molecular force field with RMSD of crystallographic heavy atoms kept at 0.30 Å. A grid box was generated to define the centroid of the active site. All the compounds were docked into the catalytic pocket of SARS CoV-2 main protease by using the Glide module of Schrödinger suite 2019-4 in extra precision (XP) mode [46]. The ligands with significant Glide scores have more binding affinity towards SARS CoV-2 main protease enzyme. To predict the free energy of binding for the set of ligands in complex with a receptor, post-docking energy minimization studies were performed using Prime molecular mechanics-generalized Born surface area (MM-GB/SA) of Schrödinger 2019-4. The energy for minimized XP docked pose of ligand-receptor complex was calculated using the OPLS3 force field and generalized Born/surface area (GB/SA) continuum VSGB 2.0 solvent model [47, 48].

## Results

**Fig. 1:**
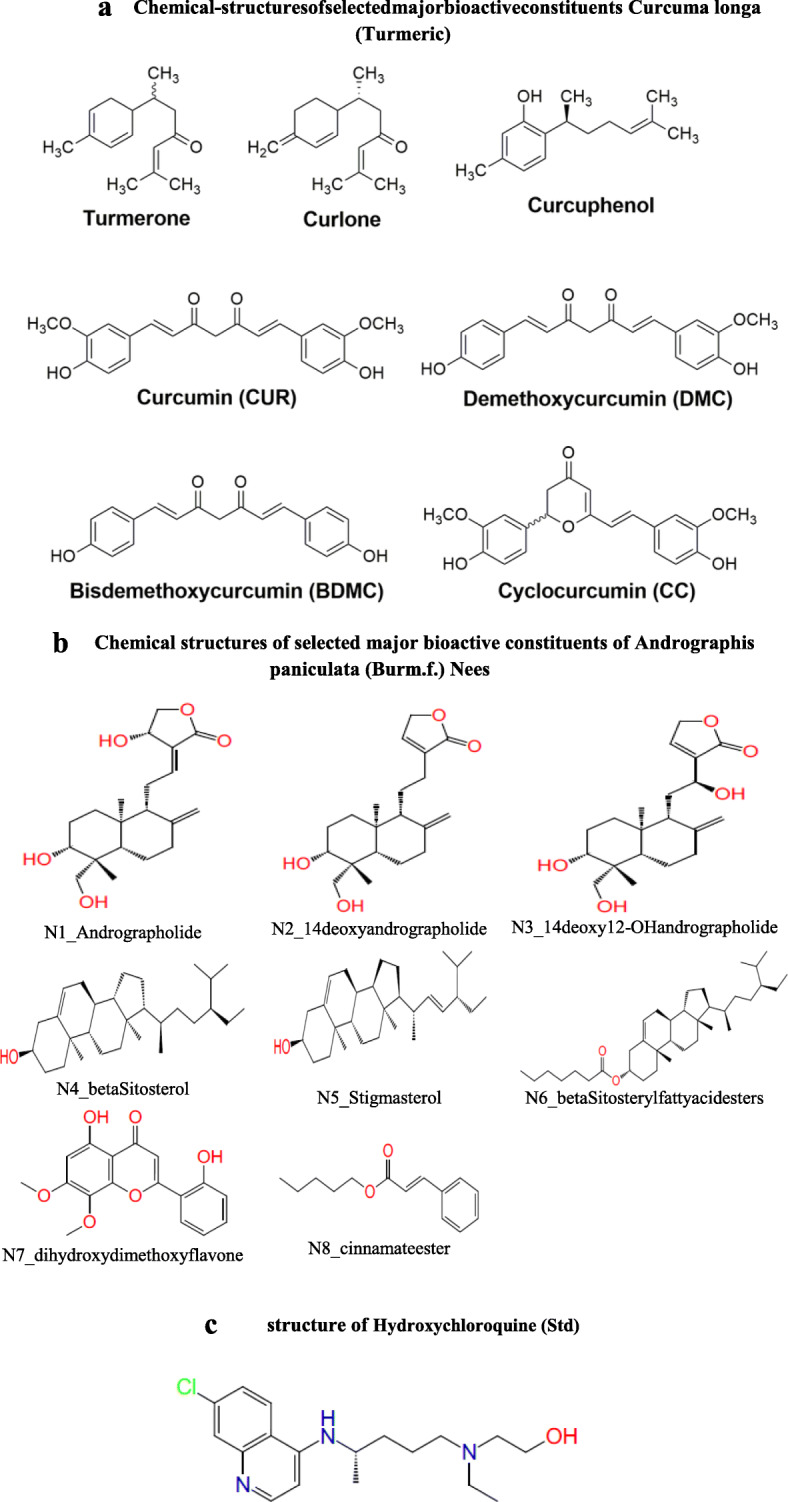
Structures of phytochemical constituents. **a** Chemical structures of selected major bioactive constituents of *Curcuma longa* (turmeric). **b** Chemical structures of selected major bioactive constituents of *Andrographis paniculata* (Burm.f.) Nees. **c** Structure of hydroxychloroquine (Std)

**Fig. 2:**
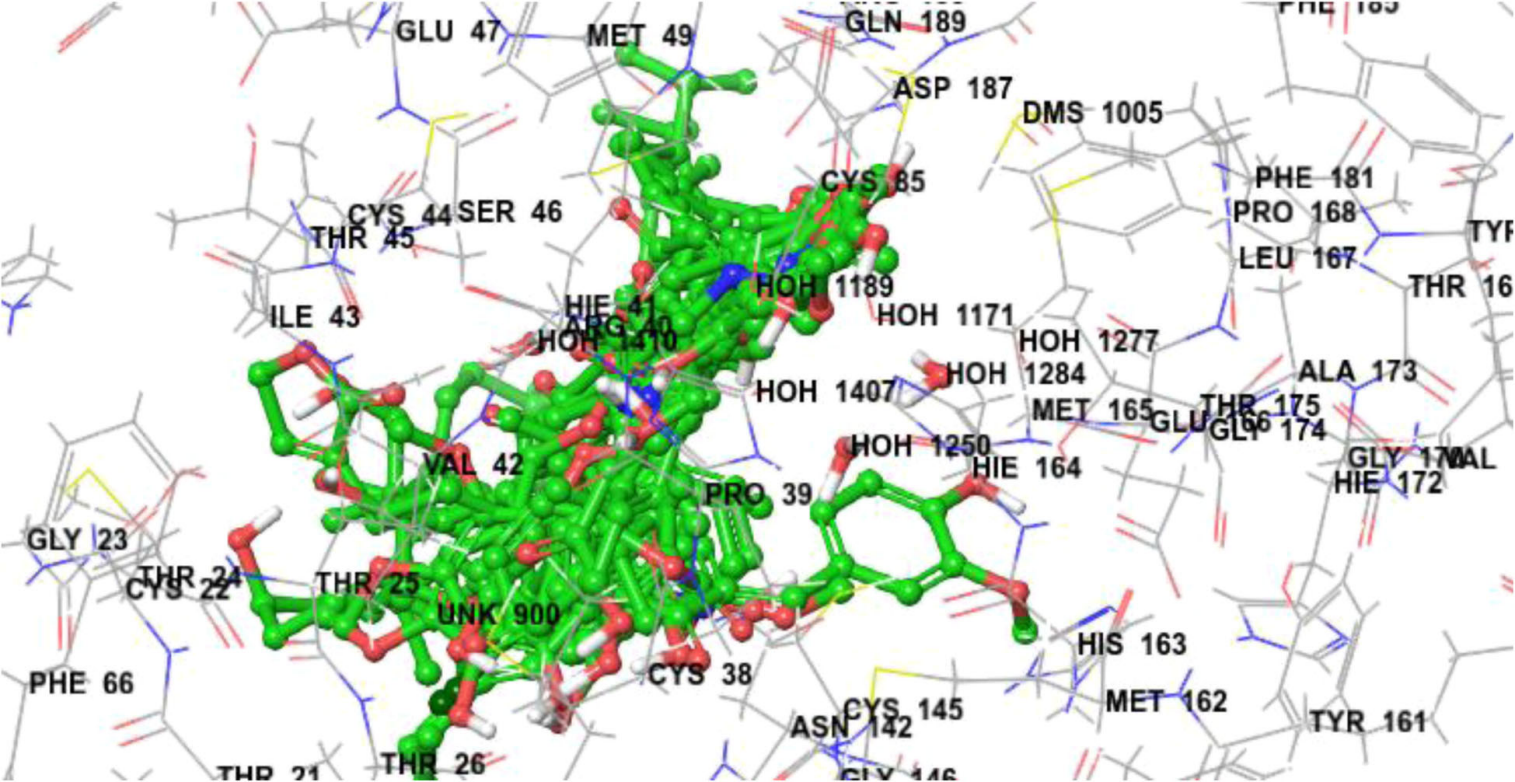
Docked poses of all compounds with SARS CoV-2 main protease (5R82)

**Fig. 3:**
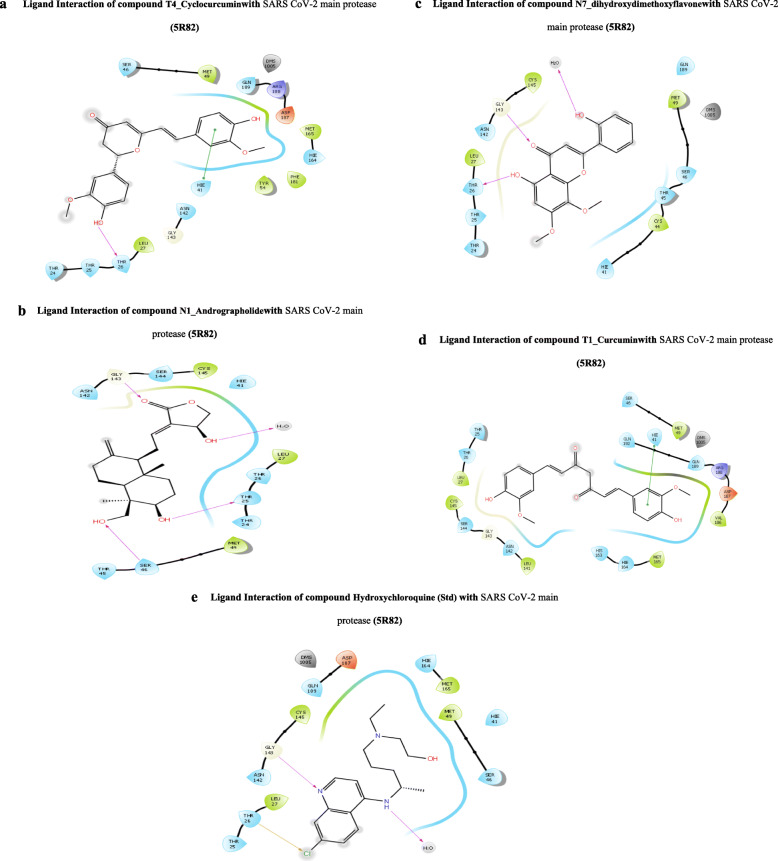
**a** Ligand interaction of compound T4_Cyclocurcuminwith SARS CoV-2 main protease (5R82). **b** Ligand interaction of compound N1_Andrographolidewith SARS CoV-2 main protease (5R82). **c** Ligand interaction of compound N7_dihydroxydimethoxyflavonewith SARS CoV-2 main protease (5R82). **d** Ligand interaction of compound T1_Curcumin with SARS CoV-2 main protease (5R82). **e** Ligand interaction of compound hydroxychloroquine (Std) with SARS CoV-2 main protease (5R82)

**Fig. 4:**
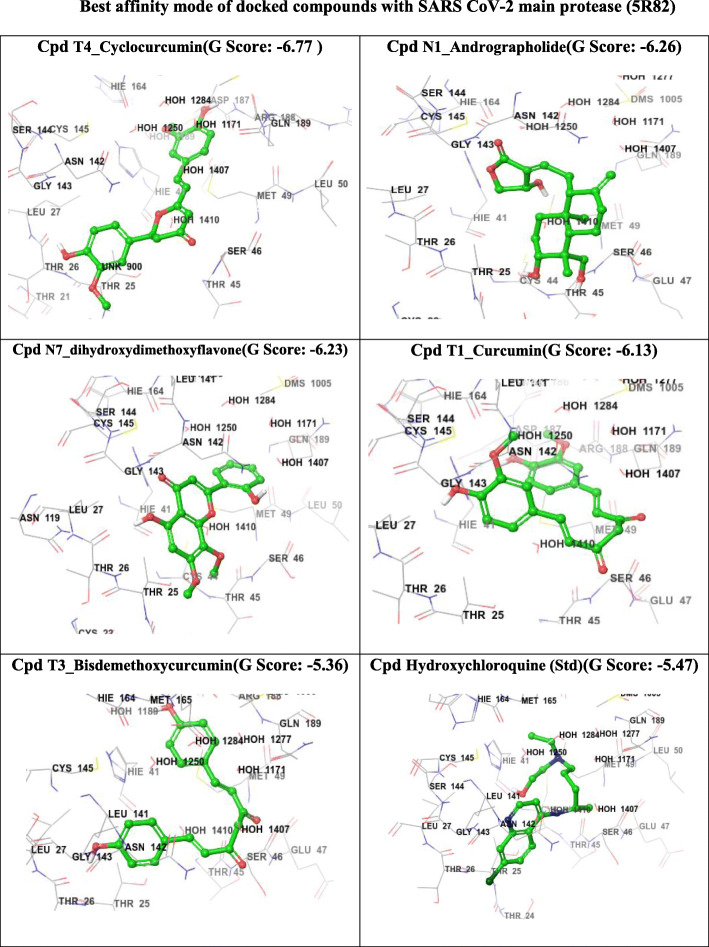
Best affinity mode of docked compounds with SARS CoV-2 main protease (5R82)

**Fig. 5:**
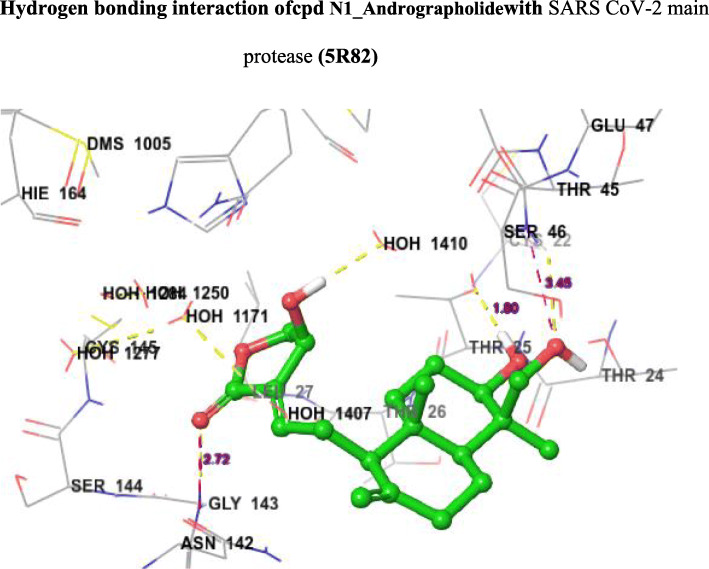
Hydrogen bonding interaction of cpd N1_Andrographolide with SARS CoV-2 main protease (5R82)

**Fig. 6:**
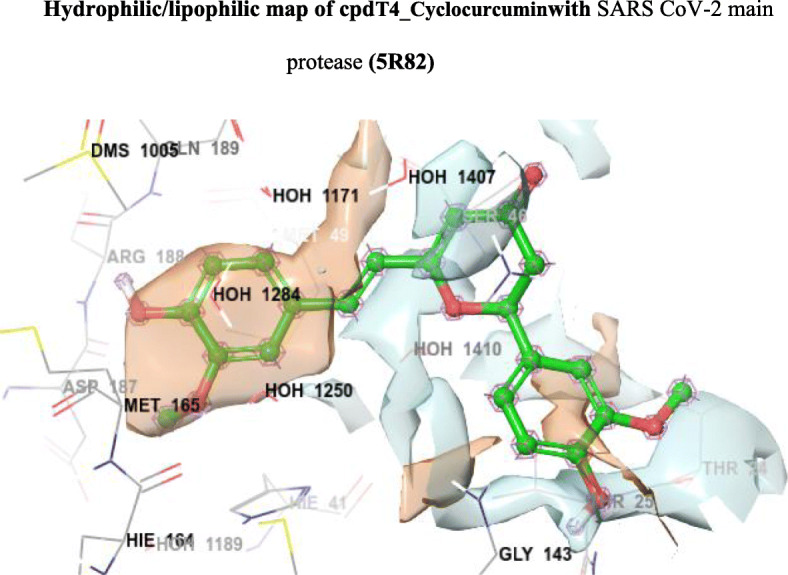
Hydrophilic/lipophilic map of cpd T4_Cyclocurcumin with SARS CoV-2 main protease (5R82)

**Fig. 7:**
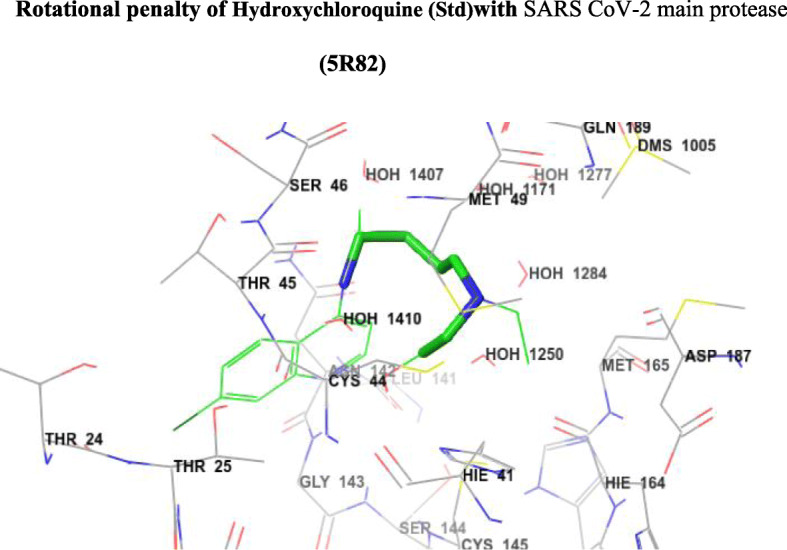
Rotational penalty of hydroxychloroquine (Std) with SARS CoV-2 main protease (5R82)

Results are summarized in Tables 1, 2, and 3 and Figs. 1, 2, 3, 4, 5, 6, and 7. The results revealed that the SARS CoV-2 main protease inhibitory property of the compounds isolated from some natural products like *Curcuma longa* (turmeric) and *Andrographis paniculata* greatly depended on the chemical nature of the substituents. The chemical structures of selected major bioactive constituents of *Curcuma longa* (turmeric) and *Andrographis paniculata* are given in Fig. 1a and b. The anti-malarial drug which was currently recommended in many countries like the USA, India, etc. [49] for the treatment of COVID-19 is hydroxychloroquine (Fig. 1c).

**Table 1:** Docking studies for phytochemical constituents of *Curcuma longa* (turmeric) and *Andrographis paniculata* with SARS CoV-2 main protease (5R82)

Cpd	Glide score	Lipophilic EvdW	Phob En	H bond	XP electro	Low MW	Rot Penal	XP penalties
T4_Cyclocurcumin	− 6.77	− 4.12	0	− 2.36	− 0.35	− 0.27	0.18	0
N1_Andrographolide	− 6.26	− 1.27	0	− 4.01	− 1.3	− 0.33	0.2	0
N7_dihydroxydimethoxyflavone	− 6.23	− 2.69	0	− 2.44	− 0.84	− 0.45	0.08	0
T1_Curcumin	− 6.13	− 4.14	0	− 1.46	− 0.72	− 0.27	0.37	0
T3_Bisdemethoxycurcumin	− 5.36	− 4.13	0	− 0.7	− 0.6	− 0.47	0.5	0
T2_Demethoxycurcumin	− 5.25	− 3.69	0	− 1.06	− 0.62	− 0.37	0.42	0
T7_Curcuphenol	− 5.13	− 4.34	0	− 0.7	− 0.31	− 0.5	0.6	0
N3_14deoxy12hydroxyandrographolide	− 5.11	− 2.89	0	− 2.12	− 0.27	− 0.33	0.27	0
T6_Curlone	− 3.89	− 4.05	0	0	− 0.07	− 0.5	0.6	0
N2_14deoxyandrographolide	− 3.88	− 1.96	0	− 2.07	− 0.54	− 0.39	0.29	0
T5_Turmerone	− 3.78	− 3.87	0	0	− 0.01	− 0.5	0.6	0
N8_cinnamateester	− 3.31	− 3.55	0	0	− 0.02	− 0.5	0.76	0
N5_Stigmasterol	− 2.28	− 3.6	0	0	0.02	− 0.12	0.15	1
N6_βSitosterylfattyacidesters	− 1.89	− 2.66	0	− 0.3	− 0.02	0	0.32	0
N4_betaSitosterol	− 1.36	− 1.86	0	0	− 0.06	− 0.12	0.2	0
Hydroxychloroquine (Std)	− 5.47	− 3.15	0	− 1.75	− 0.69	− 0.38	0.5	0

**Table 2:** In silico ADMET screening for phytochemical constituents of *Curcuma longa* (turmeric) and *Andrographis paniculata*

Compounds	Mol. Wt.	Dipole	Donor HB	Accpt HB	QPlog o/w	#metab	Rule of five	%Human oral absorption
T1_Curcumin	368.385	8.366	2	7	3.301	5	0	88.976
T2_Demethoxycurcumin	338.359	9.291	2	6.25	2.821	4	0	85.615
T3_Bisdemethoxycurcumin	308.333	8.477	2	5.5	2.585	3	0	81.091
T4_Cyclocurcumin	368.385	5.335	2	5.75	3.488	6	0	90.504
T5_Turmerone	218.338	3.649	0	2	4.036	6	0	100
T6_Curlone	218.338	3.147	0	2	3.991	5	0	100
T7_Curcuphenol	218.338	1.472	1	0.75	4.419	6	0	100
N1_Andrographolide	350.454	6.319	3	8.1	1.455	6	0	77.655
N2_14deoxyandrographolide	334.455	4.004	2	6.4	2.46	6	0	91.184
N3_14deoxy12OH_andrographolide	350.454	4.508	2	7.1	2.04	6	0	83.156
N4_betaSitosterol	414.713	2.542	1	1.7	7.643	3	1	100
N5_Stigmasterol	412.698	2.464	1	1.7	7.473	5	1	100
N6_βSitosterylfattyacidesters	526.885	3.304	0	2	9.625	3	2	100
N7_dihydroxydimethoxyflavone	314.294	3.726	1	4.5	2.682	4	0	93.829
N8_cinnamateester	218.295	4.054	0	2	3.983	0	0	100
Hydroxychloroquine (std)	335.876	6.854	2	5.7	3.369	5	0	93.213
**Recommended values**	130–725	1–12.5	0–6	2–20	− 2–6.5	1–8	max 4	> 80% is high < 25% is poor

*Mol. Wt.* molecular weight of the molecule, *Dipole* computed dipole moment, *Donor HB* estimated number of hydrogen bonds that would be donated by the solute to water molecules in an aqueous solution, *Accpt HB* estimated number of hydrogen bonds that would be accepted by the solute from water molecules in an aqueous solution, *QPlog o/w* predicted octanol/water partition coefficient, *#metab* number of likely metabolic reactions, *Rule of five* number of violations of Lipinski’s rule of five, *%Human oral absorption* predicted human oral absorption on 0 to 100% scale

**Table 3:** Binding free energy calculation using Prime/MM-GBSA approach

Compd	MMGBSA_dG_Bind	MMGBSA _dG_Bind_ Coulomb	MMGBSA _dG_Bind_ Covalent	MMGBSA_dG_Bind Hbond	MMGBSA _dG_Bind_ Lipo	MMGBSA_dG_Bind_vdW
T4_Cyclocurcumin	− 36.0315	− 31.6404	8.0570	− 0.1385	− 11.6120	− 28.1951
N1_Andrographolide	− 34.6766	− 28.4227	6.9893	− 1.9077	− 4.4014	− 28.5162
N7_diOHdiOMeflavone	− 50.6953	− 41.1895	1.6877	− 1.5594	− 8.5302	− 26.2631
T1_Curcumin	− 50.3408	− 18.0802	1.5801	− 2.6563	− 11.9153	− 42.2597
T3_BisdeOMecurcumin	− 43.5559	− 22.2542	− 1.599	0.5231	− 11.2505	− 35.4184
T2_Demethoxycurcumin	− 38.7071	− 9.1482	6.6291	− 1.3511	− 11.0807	− 42.2478
T7_Curcuphenol	− 26.1402	− 11.4548	14.9959	0.0274	− 17.0435	− 27.3193
N3_14deoxy12OHandrographolide	− 29.3622	− 5.8525	− 9.0786	0.1215	− 10.4797	− 33.9966
T6_Curlone	− 22.3669	− 23.7502	2.6632	− 0.3572	− 9.3057	− 17.1282
N2_14deoxyandrographolide	− 39.6148	− 17.6018	7.7512	− 1.4333	− 14.1630	− 35.8594
T5_Turmerone	− 24.1033	14.8710	− 12.9950	3.1283	− 8.2392	− 37.1469
N8_cinnamateester	− 30.7961	6.1548	1.5202	0.4830	− 10.6485	− 41.1941
N5_Stigmasterol	− 31.5416	18.6211	− 6.1436	2.5054	− 15.3476	− 41.7355
N6_βSitosterylfattyacidesters	1.1272	27.1428	− 0.7667	3.0617	− 5.4991	− 29.3366
N4_betaSitosterol	− 25.0588	− 5.7182	1.8587	1.9414	− 10.8267	− 31.8647
Hydroxychloroquine (std)	− 26.9975	− 4.9621	2.1824	0.0011	-9.2894	− 33.0622

The docking studies of the ligands to protein active sites were performed by an advanced molecular docking program Glide module of Schrodinger suite 2019 Maestro-12.2 version for determining the binding affinities of the compounds. The designed analogues were docked towards the SARS CoV-2 main protease (PDB ID 5R82) in order to ascertain their inhibitory activity. The analogues show best fit root mean square difference (RMSD) value of 0.2.

The results are summarized in Table 1. Almost all the compounds are docked in the same binding pocket.

The 2D-ligand interaction diagrams of T4_Cyclocurcumin, N1_Andrographolide, N7_dihydroxydimethoxyflavone, and T1_Curcumin with SARS CoV-2 main protease (PDB ID 5R82) are given in Fig. 3a–d. The 2D-ligand interaction diagram of hydroxychloroquine is given in Fig. 3e.

From the molecular docking study, it was revealed that the ligands have shown agreeable Glide G score values from − 6.13 kcal/mol (T1_Curcumin) to − 6.77 kcal/mol (T4_Cyclocurcumin) when compared to the currently recommended drugs for COVID-19 hydroxychloroquine (G score − 5.47) and nelfinavir (− 5.93). When compared to remdesivir (− 6.38), cyclocurcumin from turmeric is significantly more active. From the obtained binding modes, it was illustrated that the ligands formed hydrophobic interactions and hydrogen bonding interactions with different residues THR24 to GLN192 surrounding the active pocket which was shown in Fig. 4. The ligand N1_Andrographolide exhibited hydrogen bonding interaction with some amino acid residues and with some water molecules which are shown in Fig. 5. The presence of aromatic features and different heterocyclic rings majorly contributed towards lipophilic factors (Fig. 6).

The Glide score of the standard compound hydroxychloroquine was decreased because of the rotational penalty of the side alkyl chain which was shown in Fig. 7.

Molecular docking was additionally assessed with MM-GBSA free restricting vitality which is identified with the post-scoring approach for SARS CoV-2 main protease (PDB ID 5R82) target and the values are shown in Table 3.

## Discussion

From the docking results, as shown in Table 1, it is clearly demonstrated that some of the chemical constituents from turmeric like cyclocurcumin and curcumin and from *Andrographis paniculata* like andrographolide and dihydroxy dimethoxy flavone significantly bind with the active site of COVID-19 main protease with Glide score more than − 6 when compared to the currently recommended drug hydroxychloroquine (G score − 5.47) and significantly inhibit SARS CoV-2 main protease and may be active against COVID-19 on further process. The above compounds have good affinity to the receptor due to more lipophilic character and also due to hydrogen bonding. From the 2D-ligand interaction diagrams, almost all the compounds exhibited similar mode of interactions with SARS CoV-2 main protease and the residues THR24, THR25, THR26, LEU27, SER46, MET49, HIE41, GLN189, ARG188, ASP187, MET165, HIE164, PHE181, and THR54 play a crucial role in binding with ligands.

From Fig. 5, the docking score of the ligand N1_Andrographolide is increased due to hydrogen bonding interaction with SER46 (H-bond length 3.45 Å), GLY143 (H-bond length 2.72 Å), and THR25 (H-bond length 1.90 Å) residues and with some water molecules. From Fig. 6, it is clearly demonstrated that most of the aromatic features are covered in the lipophilic region (red color) which contributed towards lipophilic factors.

From Fig. 7, the Glide score of the standard hydroxychloroquine is decreased because of the rotational penalty due to the rotation of the side alkyl side chain present at the fourth position of quinoline.

From the results of MM-GB/SA studies, the dG bind values were observed in the range of − 34.6766 (N1_Andrographolide) to − 50.69 kcal/mol (N7_dihydroxy dimethoxy flavone) for significantly active compounds and also dG vdw values, dG lipophilic values, and the energies are positively contributing towards total binding energy. The accuracy of docking is confirmed by examining the lowest energy poses predicted by the scoring function. The Glide score and MM-GBSA free energy obtained by the docking of ligands into the coupling pocket are more stable.

## Conclusion

From the results of the docking study, the chemical constituents of *Curcuma longa* (turmeric) and *Andrographis paniculata* demonstrated better arrangement at a dynamic site. The in silico structuring strategy embraced in the present investigation helped for recognizing some lead molecules and furthermore may somewhat clarify their useful impact for further determinations like in vitro and in vivo assessments. Results from the in silico study exhibited that many of the chemical constituents from *Curcuma longa* (turmeric) and *Andrographis paniculata* family may be useful against COVID-19 by inhibiting SARS CoV-2 main protease enzyme. Based on in silico studies, the chemical constituents such as cyclocurcumin and curcumin from turmeric and andrographolide and dihydroxy dimethoxy flavone from *Andrographis paniculata* are significantly active against COVID-19 by inhibiting SARS CoV-2 main protease enzyme with remedial possibilities and are probably going to be helpful after further refinement. In conclusion, consuming turmeric in our diet regularly may be a useful remedy in the prevention of the coronavirus.

## Data Availability

All data and material are available upon request.

## Abbreviations

COVID-19: Coronavirus disease 2019
MM-GBSA: Molecular mechanics-generalized Born surface area
PDB: Protein data bank
OPLS3: Optimized potentials for liquid simulations
XP: Extra precision
